# Editorial: Precision Physical Activity and Exercise Prescriptions for Disease Prevention: The Effect of Interindividual Variability Under Different Training Approaches

**DOI:** 10.3389/fphys.2019.00646

**Published:** 2019-05-24

**Authors:** Robinson Ramírez-Vélez, Mikel Izquierdo

**Affiliations:** Department of Health Sciences, Navarrabiomed, CIBER of Frailty and Healthy Aging (CIBERFES), Instituto de Salud Carlos III, IdiSNA, Public University of Navarra, Pamplona, Spain

**Keywords:** non-communicable chronic diseases (NCDs), precision physical activity, interindividual variability, responders in clinical trials, high-intensity interval training (HIIT), cardiovascular training, resistance training (RT)

## Introduction

Optimizing exercise training to improve health biomarkers and reduce the risk of chronic disease and premature mortality is imperative to long-term health (Ross et al., 2016; Ramírez-Vélez et al., 2017). Indeed, in the era of “precision medicine,” it is reasonable to assume that the prescription of exercise as a treatment modality should be individually tailored to the specific characteristics of the patient with respect to program variables. This Research Topic consists of 11 articles, of which nine contain original data, one is a longitudinal study and two are review/opinion articles.

## Review/Opinion and Epidemiology Articles

Solomon's review lays the groundwork of the Research Topic, discussing the evidence and, perhaps more importantly, documenting the origins of inter-individual variability for people with or at risk of diabetes, in relation to responders and non-responders. Solomon underlines the compelling need for prospective trials to identify physiological or molecular signatures that can predict inter-individual variability with regards to exercise-induced changes in blood glucose levels.

Lazarus and Harridge's opinion review claims that the shape of a person's overall performance profile with regards to age is fundamentally independent of discipline, distance, or phenotype. The authors posit that with appropriate training this same profile and trajectory, albeit with a decline in performance times, would be produced by all individuals engaging in sufficient physical activity/exercise. The authors' evidence would indicate that being physically active is by far the best approach for achieving optimal aging.

Whitney and Peterson evaluated the capacity of diverse post-processing methods of handgrip strength to predict mortality and incident cerebrovascular events in 4,143 participants aged ≥65 years from the National Health and Aging Trends Study who were followed for 6 years. Their findings suggest that the variety of post-processing methods might have differing predictive capacity in the elderly in relation to the outcome of interest. Nevertheless, because absolute handgrip measures correlated with both cerebrovascular events and mortality, they may be considered beneficial for screening in the elderly.

## Interventional Articles

Domínguez-Sanchéz et al. compared neurotrophic factor responses after one session of high-intensity interval training (HIIT), resistance training (RT), or both, in a group of overweight and physically inactive adults aged 18–30 years. The main finding was that compared with baseline levels, all three protocols provoked greater changes in neurotrophin levels. Also, the combined (HIIT + RT) and RT regimens elicited greater changes in the levels of brain-derived neurotrophic factor, neurotrophin-3, and neurotrophin-4/5 than the HIIT regimen alone.

Lewis et al. showed for the first time that genetic variability of the acid ceramidase gene has a significant effect on exercise tolerance and achievement of an exercise program. Given the recognized health benefits of regular exercise, assessing individual exercise potential influenced by genetic variation before starting an intervention may be useful in maximizing exercise adherence and, therefore, the potential health benefits. These results reinforce the idea that individual differences in biological response to exercise exposure are heritable, with estimates ranging from 29 to 70%.

Valenzuela et al. illustrate the beauty and complexity of effects of a 14-week intradialytic combined exercise (aerobic + RT) protocol on patients' mental and health status. Their data suggest that intradialytic combined exercise benefits were observed for 6-min walk test, 10-repetition sit-to-stand performance time and handgrip strength. These results are of major clinical importance, as they suggest the benefits of exercise in dialytic patients, albeit with the assumption that these benefits represent the response of the majority of individuals to potentially reduce morbimortality risk in these patients.

Plyometric jump training (PJT) has received increasing attention in recent years as a modality to increase muscular strength, particularly in the legs. Ramirez-Campillo et al. present the physical fitness responses to work-matched soccer training vs. PJT performed using a novel training regimen, which allows improved individualization of training approaches.

Álvarez et al. reported improvements in obesity markers, metabolic risk factors, and endurance performance in both prehypertensive and normotensive groups. Also, these changes in blood pressure were accompanied by other acknowledged improvements of HIIT for body composition and metabolic and endurance performance, in both study cohorts. These authors suggest that to normalize high blood pressure and improve lipid profiles, an appropriate type of exercise training must be chosen. In addition, other non-pharmacological strategies are required to prevent hypertension.

Myokines and exercise-induced proteins/hormones are known to play important roles in a variety of physiological functions. In this context, He et al. compared the response following one session of HIIT or RT on the levels of several myokines/hormones involved in metabolic function, at baseline and 0, 1, 3, 24, 48, and 72 h post-exercise in 17 healthy, non-athletic men (23 ± 3 years). With the exception of fibroblast-growth factor-21, no overall differences were found in the myokine response to HIIT or RT. However, the authors observed considerable interindividual variability, with some subjects specifically responding to some but not other training session types.

Naves et al. studied and compare the effects of two different 8-week interval-training programs—SIT (23-min) and HIIT (33-min)—on anthropometric parameters and cardiorespiratory fitness in healthy young women, in 49 young active women with a mean age 30.4 ± 6.1 years. Their main findings were that both protocols led to improvements in anthropometric parameters and also cardiorespiratory fitness, even in the absence of changes to dietary intake. Moreover, greater reductions in the sum of skinfolds were seen after the SIT protocol as compared with HIIT.

With personalized medicine becoming increasingly widespread, Williams et al. compared, in a large sample of subjects (*n* = 677) from different laboratories (18 centers), the experiential rates of likely responders in an assortment of aerobic training interventions. Interestingly, the authors found that studies with the shortest duration and high-volume training loads often had the least significant gains and fewer clinically meaningful VO_2_peak responders. Because, many of the benefits of exercise are closely associated with improvements in CRF, as determined by peak or maximal oxygen consumption (VO_2_peak/VO_2_max), the authors suggest that future large, well-controlled studies with comparator groups and cross-over designs may help to identify influential variables and the ideal training load for $\dot{\text{V}}$O_2_peak trainability.

## Perspectives

Papers in this Research Topic highlight the notion that personalized exercise is a feasible and effective lifestyle modification strategy, for all individuals with, or at risk of, non-communicable chronic diseases (DiMenna and Arad, 2018). Accordingly, more research is needed to compare the training paradigms in defined subgroups, for instance, at-risk subjects, and at different stages of disease join to the point. With many issues unresolved, further research is warranted before exercise can conscientiously be prescribed as “precision medicine” to address cardiometabolic risk factors and their progression to many non-communicable diseases.

## Author Contributions

RR-V and MI: drafted the manuscript. All authors approved the final version.

### Conflict of Interest Statement

The authors declare that the research was conducted in the absence of any commercial or financial relationships that could be construed as a potential conflict of interest.
